# TLR7/8 in the Pathogenesis of Parkinson’s Disease †

**DOI:** 10.3390/ijms21249384

**Published:** 2020-12-09

**Authors:** Michela Campolo, Alessia Filippone, Carmelo Biondo, Giuseppe Mancuso, Giovanna Casili, Marika Lanza, Salvatore Cuzzocrea, Emanuela Esposito, Irene Paterniti

**Affiliations:** 1 Department of Chemical, Biological, Pharmacological and Environmental Sciences, University of Messina, Viale Ferdinando Stagno D’alcontres, 31-98166 Messina, Italy; campolom@unime.it (M.C.); afilippone@unime.it (A.F.); gcasili@unime.it (G.C.); mlanza@unime.it (M.L.); salvator@unime.it (S.C.); eesposito@unime.it (E.E.); 2 Metchnikoff Laboratory, Department of Human Pathology and Medicine, University of Messina, 31-98166 Messina, Italy; carmelo.biondo@unime.it (C.B.); giuseppe.mancuso@unime.it (G.M.); 3 Department of Pharmacological and Physiological Science, Saint Louis University School of Medicine, St. Louis, MO 63104, USA

**Keywords:** TLR7, TLR8, TLR9, Parkinson’s disease, autoimmunity

## Abstract

Neuroinflammation and autoimmune mechanisms have a key part in the pathogenesis of Parkinson’s disease (PD). Therefore, we evaluated the role of Toll-like receptors (TLRs) as a link between inflammation and autoimmunity in PD. An in vivo model of PD was performed by administration of 1-metil 4-fenil 1,2,3,6-tetraidro-piridina (MPTP) at the dose of 20 mg/kg every 2 h for a total administration of 80/kg, both in single Knock Out (KO) mice for TLR7, TLR 8, and TLR9 and in double KO mice for TLR 7/8^-/-^. All animals were compared with WT animals used as a control group. All animals were sacrificed after 7 days form the first administration of MPTP. The genetic absence of TLR 7 and 8 modified the PD pathway, increasing the immunoreactivity for TH and DAT compared to PD groups and decreasing microglia and astrocytes activation. Moreover, the deletion of TLR7 and TLR8 significantly reduced T-cell infiltration in the substantia nigra and lymph nodes, suggesting a reduction of T-cell activation. Therefore, our result highlights a possibility that an immunotherapy approach, by using a dual antagonist of TLR 7 and 8, could be considered as a possible target to develop new therapies for Parkinson diseases.

## 1. Introduction

Parkinson’s disease (PD) is a neurodegenerative disorder characterized by a gradual damage of dopaminergic neurons in the substantia nigra pars compacta and presence of α-synuclein (α-syn)-rich cytoplasmic neuronal inclusion named Lewy bodies [1]. The etiology of PD is still unknown, but recent evidence indicates that adaptive immunity in concern with inflammation are involved in the pathogenesis of PD [2]. It has observed that the levels of activated T cells are increased in the cerebrospinal fluid (CSF), as well as in the midbrains, of patients with PD [3,4]. Moreover, a potential role for B cells in PD is also recently emerging [5]. Although B cells have not been detected in the brains of patients with PD, deposits of Immunoglobulin G (IgG) are found on dopaminergic neurons in these patients, and Lewy bodies themselves are coated with IgG [6], which suggests that dopaminergic neurons might be targeted by these immunoglobulins. Despite increasing evidence of a potential role for B cells in PD, it is unclear whether the observed changes in adaptive immunity are causal or are secondary to the CNS injury associated with its pathogenesis. Early clinical trials are under way to determine whether treatment with anti-α-synuclein monoclonal antibodies benefits patients with PD [7].

One of the well recognize pathways that promote an innate immune response is mediated by Toll-like receptors (TLRs) activation [8].

Most TLRs are extracellular, recognizing their ligands on the cell surface. On the other hand, nucleic acid specific TLRs, including TLR7, TLR8, and TLR9, are localized intracellularly within one or more endolysosomal compartments [9]. These TLRs are expressed on various immune cells, like plasmocytic dendritic cells, macrophage, B and T cells, monocytes, and mast cells [10,11,12,13]. Thanks to their characteristics, these endosomal TLRs may face a challenge to induce anti-pathogen immune responses while avoiding the induction of autoimmune diseases. In fact, in the context of autoimmune diseases, such as systemic lupus erythematosus (SLE), the intracellular TLRs can also be inappropriately activated by self-nucleic acids complexed with autoreactive antibodies [14,15]. Activation of TLRs may be induced by DAMPs [16,17] related to oxidative stress, such as cell debris or molecules released by damaged or dying neurons; in PD, α-synuclein itself could work as a DAMPs, activating Toll-like receptors (TLRs) [18,19]. In particular, recent papers have focused the attention only on the roles of extracellular TLR, such as 2 and 4, in the pathogenesis of PD [20,21], never looking to the involvement of the other TLRs, such as the intracellular TLR7, 8, and 9 in PD etiology.

Therefore, considering that adaptive immune system contributes to PD is an area of active research, the aim of this study was to assess the involvement of TLRs 7, 8, 9 in an in vivo model of PD induced by administration of the neurotoxin 1-metil 4-fenil 1,2,3,6-tetraidro-piridina (MPTP) in TLR 7, 8, and 9 Knock Out (KO) mice.

## 2. Results

### 2.1. Effective Role of TLR 7, 8, and 9 in the Pathogenesis of PD

**Figure 1:**
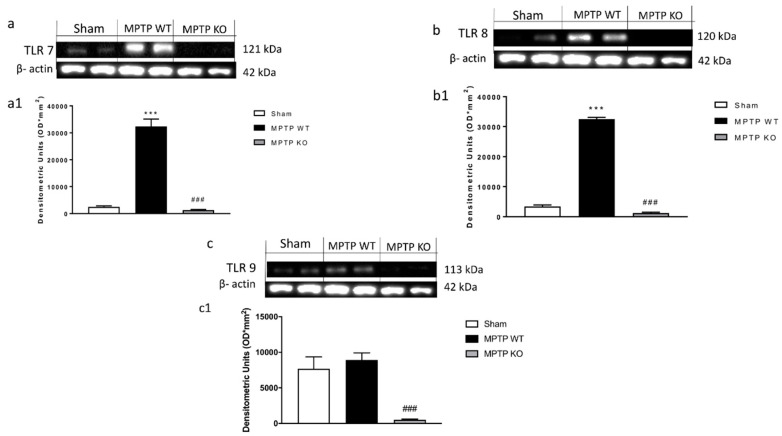
Evaluation of Toll-like receptors (TLR) 7, 8, and 9 in Parkinson’s disease (PD). Using Western Blot analysis, we evaluated the expression of TLR7, 8, 9 (**a**–**c**). Following MPTP intoxication the expression of TLR 7, 8, and 9 was significantly increased in WT mice (Figure 1(a,a1,b,b1,c,c1), respectively), no expression was evident in Knock Out (KO) mice (Figure 1(a,a1,b,b1,c,c1)). Data are expressed as Mean ± SEM from N = 10 Mice for each group. *** *p* < 0.001 vs. SHAM WT; ### *p* < 0.001 vs. SHAM WT.

To validate the effective role of endosomal TLRs during a parkinsonian event, we assessed the expression of TLR 7, 8, and 9 after MPTP induction in C57BL/6 Wild type (WT )mice. Western blot results, at the time point examined, showed a significant up-regulation of TLRs receptors 7, 8, and 9 in WT mice following MPTP administration compared to control group (Figure 1(a,a1,b,b1,c,c1) respectively); while, in the KO mice, respectively, for TLR7, 8, and 9, there was no expression of the single receptors, confirming the lack of TLR in the KO mice (Figure 1(a,a1,b,b1,c,c1) respectively).

### 2.2. Role of TLR 7, 8, and 9 and on Behavioral Impairments

**Figure 2:**
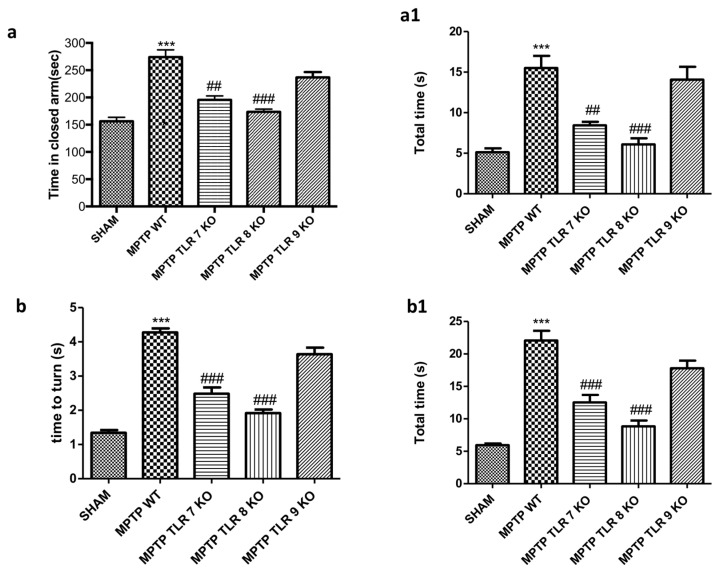
Effect of TLR 7, 8, and 9 on behavioral impairments induced by 1-metil 4-fenil 1,2,3,6-tetraidro-piridina (MPTP) intoxication. Elevated plus-maze (EPM) test showed that MPTP WT mice spent more time in the closed arms (**a**,**a1**), while TLR 7 and 8 KO mice increased the time to stay in the open arms (**a**,**a1**). There were not any significant improvements in TLR9 KO mice (**a**,**a1**). Anxiety is expressed as mean of total number of arm entries in the open arms and number of closed-arm entries (**a1**). Each Data are means ± SEM of 10 mice for each group. *** *p* < 0.001 vs. SHAM WT, ### < 0.001 vs. MPTP WT; ## < 0.01 vs. MPTP WT. “Time to turn” and “total time” increased in MPTP in WT mice (**b**,**b1**), while TLR7 and 8 KO mice showed a significant reduction (**b**,**b1**). TLR 9 KO mice wasn’t able to affect motor damage following MPTP (**b**,**b1**). Each Data are means ± SEM of 10 mice for each group. *** *p* < 0.001 vs. SHAM WT, ### < 0.001 vs. MPTP WT.

Behavioral deficits, and specifically motor activity, in PD are critical to evaluate the extent of DA neuron degeneration. To evaluate anxiety, we used anxiety-like behavior in the elevated plus-maze (EPM). The behavioral test that we performed indicated an important increase in the percentage of time spent in the closed arms of MPTP WT mice compared to the SHAM group (Figure 2(a,a1)).

Instead, TLR 7 and 8 KO mice showed a reduction of the time spent in the closed arms compared to MPTP WT group (Figure 2(a,a1)). There were not any significant improvements in TLR9 KO mice compared with MPTP WT-injured mice (Figure 2(a,a1)). Moreover, to evaluate the alteration on motor function induced by MPTP administration, we assessed the pole test as measure of bradykinesia. We observed that “Total time” and “time to turn” significantly increased after injection of MPTP in WT mice compared to control group (Figure 2(b,b1)), whereas TLR7 and 8 KO mice showed a significant decrease of “total time” and “time to turn”, suggesting a significant reduction of bradykinesia (Figure 2(b,b1)); TLR 9 KO mice were not able to ameliorate motor damage following MPTP administration (Figure 2(b,b1)).

### 2.3. Role of TLR 7, 8, and 9 on Dopaminergic Neurons Lost

**Figure 3:**
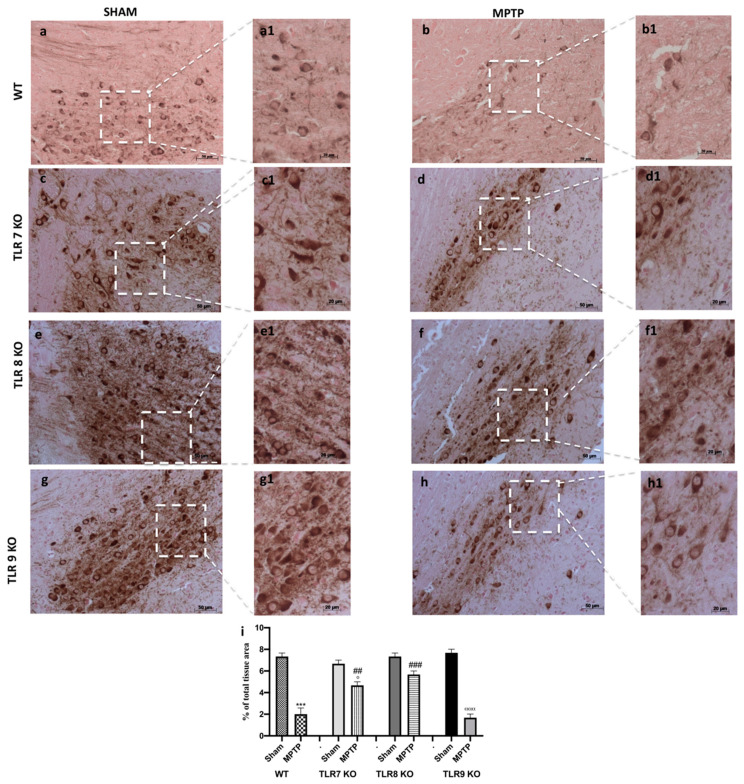
Effect of TLR 7, 8, and 9 on TH immunoreactivity. By immunohistochemistry, we observed a TH immunoreactivity. WT mice MTPT-injected (**b**,**b1**) revealed a significant decrease of TH+ neurons compared to SHAM WT (**a**,**a1**). In TLR7 and 8 KO mice the reduction of DA neurons was significantly preserved (**d**,**d1**,**f**,**f1**, respectively), compared to, respectively, SHAM groups (**c**,**c1**,**e**,**e1**, respectively), while, in TLR 9 KO mice subject to MPTP, there was a reduction of TH positive cells (**h**,**h1**) compared to TLR9 SHAM (**g**,**g1**). Panel A and B showed WT mice. See densitometric analysis (**i**). Data are means ± SEM of 10 mice for each group. *** *p* < 0.001 vs. SHAM WT ° *p* < 0.05 vs. SHAM TLR7 KO; ^ααα^ *p* < 0.001 vs. SHAM TLR9 KO; ### *p* < 0.001 vs. MTPT WT and ## *p* < 0.01 vs. MTPT WT.

Parkinson’s disease (PD) can be considered as a TH [22] deficiency syndromes of the striatum; so, to determine the role of TLR 7, 8, and 9 on DA neurons’ death, midbrain sections of single KO mice were stained for TH immunoreactivity. An important loss of TH-positive cells was detected in TLR 9 KO mice subject to MPTP administration compared to MPTP WT mice (Figure 3(h,h1,b,b1); see densitometric analysis i), while, in the TLR 7 and 8 KO mice, the loss of DA neurons was greatly reduced (Figure 3(d,d1,f,f1); see densitometric analysis i), compared to their, respectively, SHAM KO animals (Figure 3(c,c1,e,e1); see densitometric analysis i), indicating that these receptors protected from TH neuronal lost much more than MPTP WT mice (Figure 3(b,b1); see densitometric analysis i).

### 2.4. TLR7/8 Protected from MPTP-Induced Behavioral Impairments

**Figure 4:**
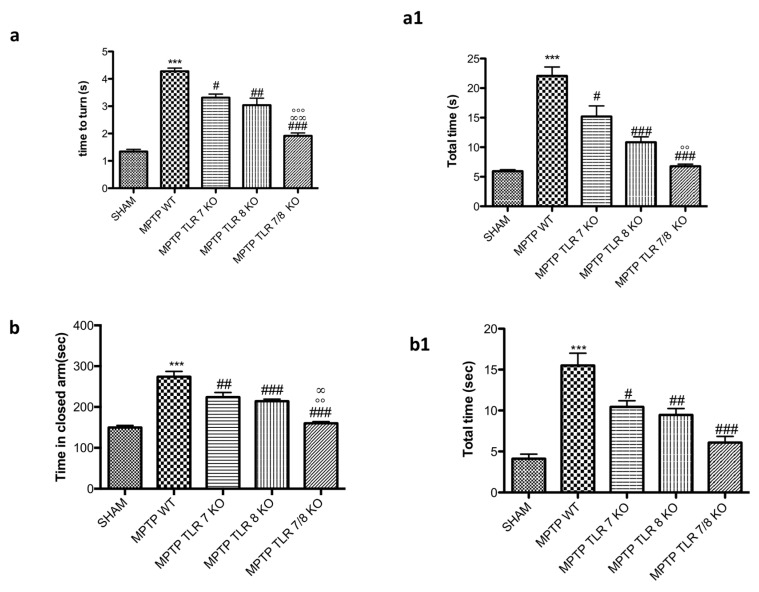
Effect of TLR7/8 on behavioral alteration induced by MPTP. “Time to turn” and “Total time” were significantly increased after injection of MPTP in WT mice compared with the control group (**a**,**a1**); TLR7 and 8 KO mice showed a reduction of both “time to turn” and “total time” (**a**,**a1**), with a significant reduction in the double TLR7/8 KO mice (**a**,**a1**). Values are mean ± SEM of 10 mice for each group. *** *p* < 0.001 vs. SHAM WT; # *p* < 0.05 vs. MPTP WT; ## *p* < 0.01 vs. MPTP WT; ### *p* < 0.001 vs. MPTP WT; °° *p* < 0.01 vs. MPTP TLR7KO; °°° *p* < 0.001 vs. MPTP TLR7KO; ∞ ∞ *p* < 0.01 vs. MPTP TLR8 KO. The effect of the absence of receptors TLR7 and 8 on anxiety behaviors was evaluated by elevated plus maze (EPM) test (**b**,**b1**). Anxiety was expressed as mean total entries in the open arms and number of entries in open arms. MPTP mice stand more time in the closed arms compared to SHAM animals (**b**,**b1**), whereas TLR7 and 8 KO mice and much more TLR 7/8 KO mice showed a reduction of time spent in closed arms (**b**,**b1**). Data are means ± SEM of 10 mice for each group. *** *p* < 0.001 vs. SHAM WT; # *p* < 0.05 vs. MPTP WT; ## *p* < 0.01 vs. MPTP WT; ### *p*< 0.001 vs. MPTP WT; °° *p* < 0.01 vs. MPTP TLR7KO; ∞ *p* < 0.05 vs. MPTP TLR8 KO.

To better understand the role of TLR7 and 8 in the behavioral impairments, we compared the single KO mice for TLR7 and 8 with the double TLR7/8 KO mice. As shown before, “total time” and “time to turn” significantly increased in MPTP WT mice (Figure 4(a,a1)); whereas TLR7 and 8 KO mice showed a reduction of “total time” and “time to turn” (Figure 4(a,a1)), the double KO TLR7/8 decreased both times much more than single KO (Figure 4(a,a1)), suggesting a substantial reduction of bradykinesia. Moreover, we evaluated emotional state by EPM test, and TLR 7/8 KO mice showed a reduction of the time spent in the closed arms compared to MPTP WT group (Figure 4(b,b1)) and to single TLR KO mice (Figure 4(b,b1)).

### 2.5. TLR7/8 Absence Reduced Loss of TH Expression

**Figure 5:**
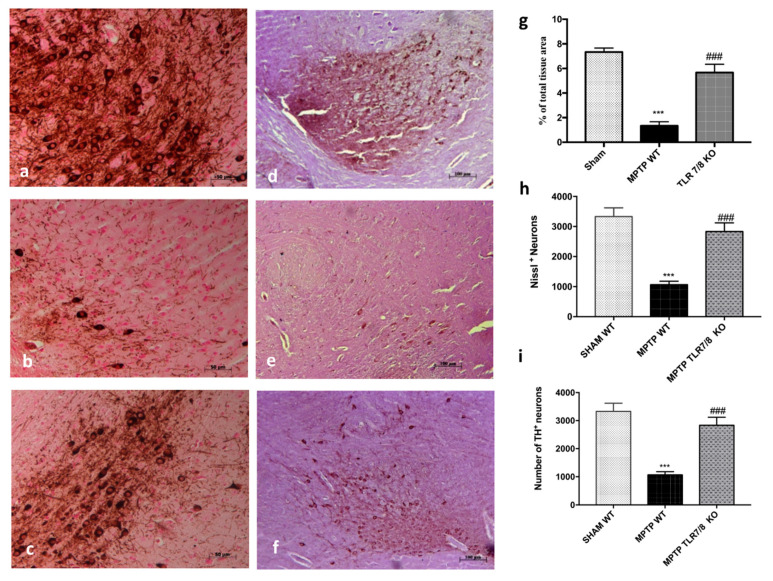
Effect of TLR7/8 on TH expression. Immunohistochemical analysis of midbrain obtained from WT mice after MPTP intoxication revealed a marked loss of TH-positive cells (**b**; see densitometric analysis, **g**) compared with SHAM (**a**; see densitometric analysis, **g**). TLR7/8 KO mice, subjected to MPTP, revealed an increase of positive staining for TH (**c**; see densitometric analysis, **g**). Data are expressed as a percentage of total tissue area and are means ± SE of 10 mice/group *** *p* < 0.001 vs. SHAM; ### *p* < 0.001 vs. MPTP WT. Stereological counting of TH-positive and cresyl-violet positive neurons in sections of the SN from one hemisphere (**i**,**h**, respectively). Representative images of TH immunohistochemistry of midbrain sections and counterstained with cresyl violet (**d**–**f**). Each data are expressed as a number of TH+ and Nissl+ neurons and are mean ± SEM from N = 10 mice/group. *** *p* < 0.001 vs. SHAM; ### *p* < 0.001 vs. MPTP WT (**h**,**i**).

In order to confirm the involvement of TLR7/8 on the dopamine pathway we evaluated, by immunohistochemical staining, the expression of TH. In the Substantia nigra (SN) of WT mice at 8 days after MPTP administration, a significant loss of TH-positive cells was clearly demonstrated (Figure 5b; see relative densitometry analysis g), while the absence of TLR7/8 considerably reduced the loss of TH-positive neurons after MPTP administration (Figure 5c; see relative densitometry analysis g). Moreover, unbiased stereology of nigral TH-positive neurons performed both in WT mice, as well as in TRL7/8 KO mice, 8 days after MPTP intoxication and showed significant neuroprotection in TLR7/8 KO. We observed a significantly decline in the number of TH-positive neurons in mice after MPTP injection, compared to SHAM groups (Figure 5d,e, respectively). This loss decreased in KO mice (Figure 5f). Similarly, Nissl-stained neurons were depleted significantly in WT mice by MPTP lesioning but not in TLR 7/8 KO mice (Figure 5h,i).

### 2.6. TLR7/8 Absence Prevented Dopamine Depletion from MPTP Toxicity

**Figure 6:**
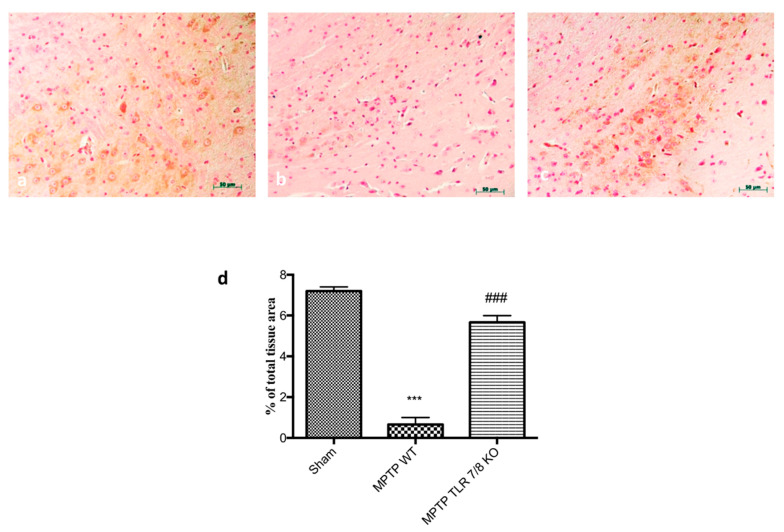
Effect of TLR7/8 on DAT expression in SN of MPTP-treated mice. Immunohistochemical analysis of midbrain obtained from WT mice after MPTP intoxication revealed an important loss of DAT-positive cells (**b**; see densitometric analysis **d**) compared to WT (**a**, see densitometric analysis **d**); while the absence of TLR7/8 preserved the reductions of DAT (**c**; see densitometric analysis **d**). Data are means ± SEM of 10 mice for each group. *** *p* < 0.001 vs. SHAM WT; ### *p* < 0.001 vs. MPTP WT.

Moreover, being that the loss of DAT is a significant sign of PD, we evaluated by immunohistochemical staining the expression of DAT. We assessed that MPTP injection induced an important reduction of DAT positive staining (Figure 6b; see densitometric analysis d) in WT mice compared to SHAM group (Figure 6a; see densitometric analysis e), while the absence of TLR7/8 preserved the severe reductions of DAT positive staining (Figure 6c; see densitometric analysis d).

### 2.7. TLR7/8 Absence Preserved α-Synuclein Accumulation in TH^+^ Neurons from MPTP Toxicity

Since α-Synuclein is a key marker in PD pathology, we evaluated the α-synuclein accumulation in the dopaminergic neurons with a double staining between TH (green) and α-synuclein (red).

**Figure 7:**
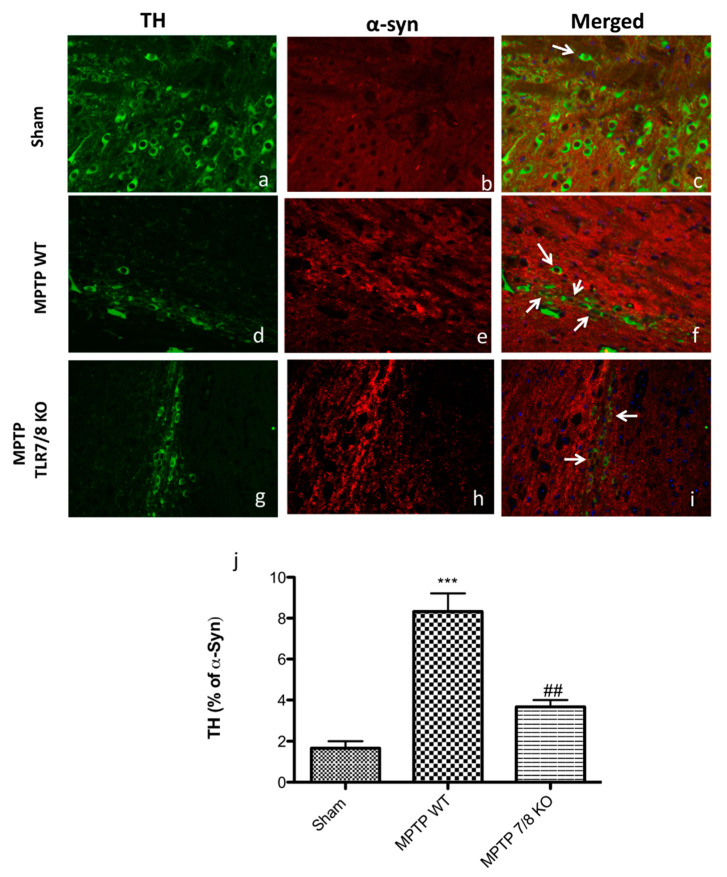
Co-localization of TH/α-synuclein after MPTP-intoxication. Results are shown for SHAM (**a**–**c**), mice after MPTP-intoxication in WT mice (**d**–**f**) and after MPTP-intoxication in KO mice (**g**–**i**) and percentage of TH positive profiles that co-labeled alpha-syn (**j**). Midbrain sections were double stained with antibodies against TH (**a**,**d**,**g**—green)/α-syn (**b**,**e**,**h**—red). Midbrain sections revealed an important expression of α-syn in dopaminergic neurons (white arrows) in the MPTP WT mice (**f**; see graphic **j**), compared with SHAM group (**b**; see graphic **j**). TH/α-syn, double stained (white arrows), was significantly decreased in MPTP TLR7/8 KO mice (**i**; see graph **j**). The white arrow represents the TH+ neurons that co-labeled with α- syn (**c**,**f**,**i**). Data are means ± SEM of 10 mice for each group. *** *p* < 0.001 vs. SHAM WT; ## *p* < 0.01 vs. MPTP WT.

A marked increase in α-syn accumulation in TH^+^ neurons was observed in the SN of WT mice after MPTP injection (Figure 7d–f; see graphic j), compared to SHAM mice (Figure 7a–c; see graphic j), while, in the TLR7/8 KO mice, we noticed a significantly decreased of α-synuclein levels into the dopaminergic neurons, indicating that the absence of TLR 7/8 preserved α-syn accumulation in TH+ neurons (Figure 7g–I; see graphic j).

**Figure 8:**
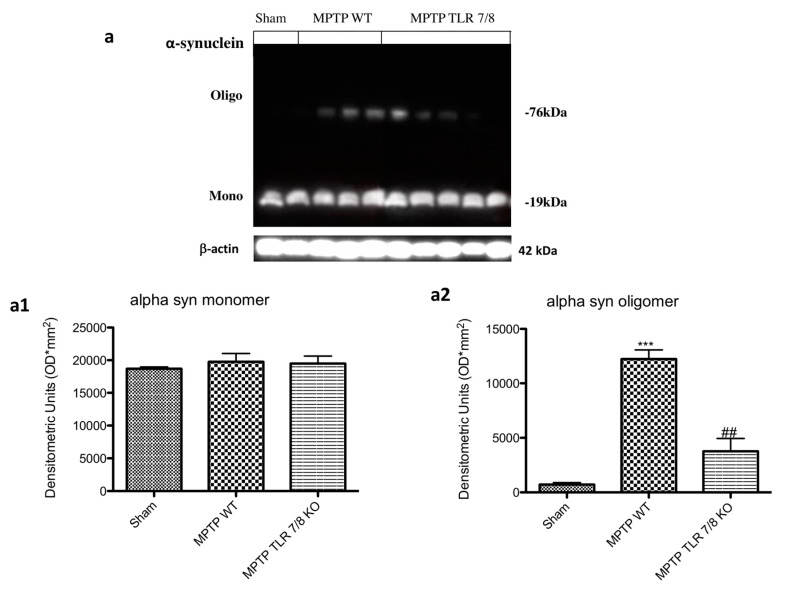
Effect of TLR7/8 on α-synuclein expression. Western blot analysis revealed an increase of α-synuclein in MPTP WT-injured mice (**a**; see densitometry analysis **a1**,**a2**). The absence of TLR 7/8 preserved mice from α-synuclein aggregates formation (**a**; see densitometry analysis **a1**,**a2**). Data are means ± SEM of 10 mice for each group. *** *p* < 0.001 vs. SHAM WT; ## *p* < 0.01 vs. MPTP WT.

Moreover, western blot analysis revealed a threefold increase of α-synuclein monomer, and oligomer in MPTP WT-injured mice (Figure 8a; see densitometry analysis a1,a2) compared with the baseline constant expression of α-synuclein in control mice. The absence of TLR 7/8 preserved mice from α-synuclein aggregates formation (Figure 8a; see densitometry analysis a1,a2).

### 2.8. Effect of TLR7/8-Receptor Absence on Expression of GFAP, IBA1, and TNFα following Treatment with MPTP

**Figure 9:**
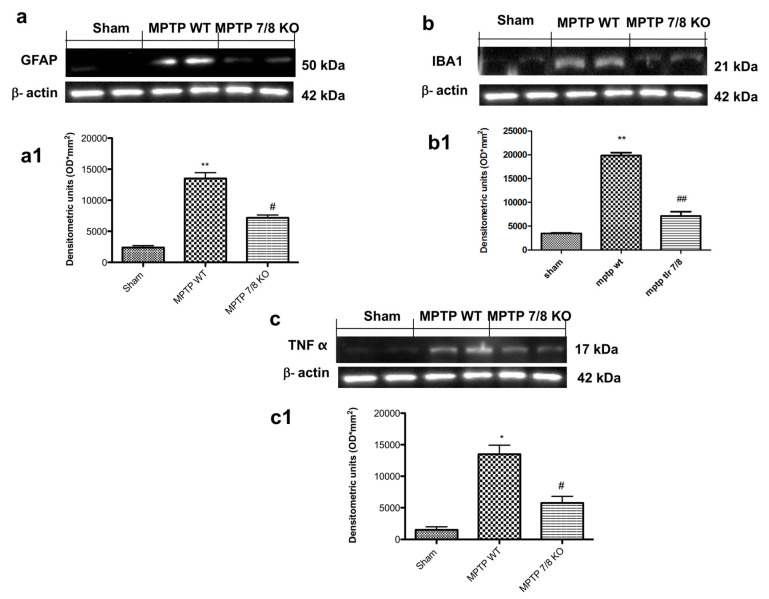
Role of TLR7/8 on GFAP, IBA1, and TNF-α expression after MPTP administration. Western blot analysis showed significant expression levels of GFAP and IBA1 in SN of WT MPTP-treated mice, which were reduced in MPTP TLR7/8 KO group (**a**,**a1**,**b**,**b1**, respectively). Western blot analysis also showed an upregulation of TNF-α expression in WT MPTP -treated mice compared to SHAM (**c**,**c1**). This expression was notably reduced in TLR7/8 KO MPTP- treated mice (**c**,**c1**). Each data are expressed as Mean ± SEM from N = 10 Mice for each group. * *p* < 0.05 vs. SHAM WT; ** *p* < 0.01 vs. SHAM WT; # *p* < 0.05 vs. MPTP WT; ## *p* < 0.01 vs. MPTP WT.

The auto-aggressive process of autoimmune PD would be enhanced by a Neuromelanin (NM)-triggered activation of microglia, leading to an amplification of the adaptive immune response against NM and the local reactivation of immigrating effector T cells [2]. The expression of GFAP and IBA1 was assessed by Western blot analysis. The results showed a basal expression of GFAP and IBA1 in SHAM groups, while astrogliosis and microgliosis markedly increased following MPTP injection in WT mice and was notably reduced in TLR7/8 KO MPTP-treated mice (Figure 9(a,a,b,b1), respectively; see densitometry analysis a1,b1). Moreover, the expression of TNFα was significantly increased in MPTP WT group and significantly reduced in the MPTP TLR7/8 KO group (Figure 9c; see densitometry analysis c1).

### 2.9. Decrease in Brain-Infiltrating Immune Cells in TLR 7/8 KO Mice after MPTP Injection

**Figure 10:**
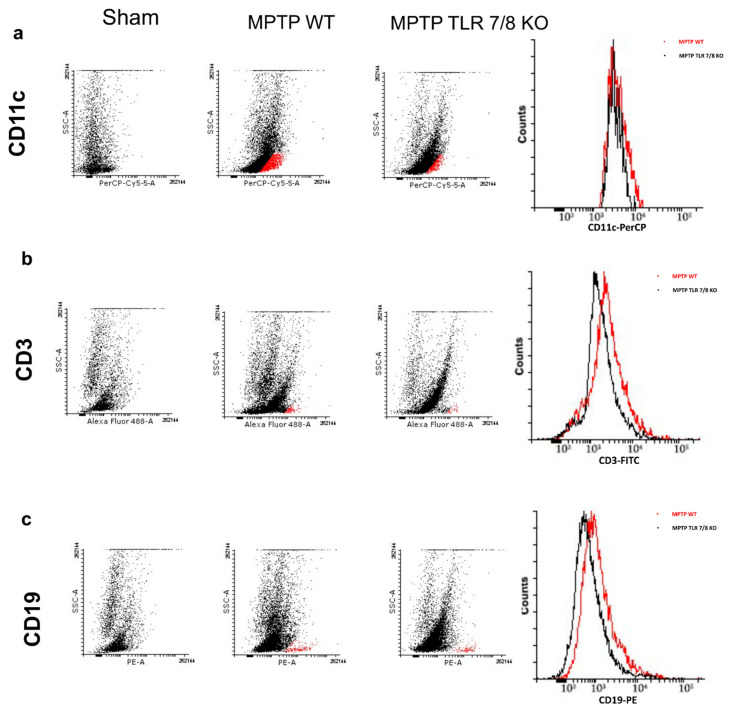
Effect of TLR7/8 on CD11c and CD3 expression. We analyzed CD11c+ and CD3+ expression by flow cytometry. MPTP injection increase CD11c cells in the brain of WT mice compared with the SHAM group (**a**). The absence of TLR 7/8 showed a trend to reduce CD11c cells infiltration (Panel **a**). An increase in CD3+ T cell positive numbers was observed in WT mice as compared to SHAM group (**b**), while TLR 7/8 KO mice showing a decreasing trend in CD3+ T cell (**b**). Moreover, CD19 B cell increased in the MPTP WT mice (**c**), while, in TLR 7/8 KO mice, we observed an important reduction of CD19+ B cell (**c**).

It has been demonstrated that DCs are actively recruited and accumulated to the inflammation sites in the CNS showing a direct correlation with T-cells in PD [2]; therefore, we analyzed CD11c^+^ and CD3^+^ expression by flow cytometry. MPTP injection led to a slight increase in CD11c cells in the brain of WT mice compared with the SHAM group (Figure 10a). The absence of TLR 7/8 clearly showed a trend to a reduction on DC brain infiltration (Figure 10a). Subsequently, an MPTP-driven increase in the brain CD3^+^ T cell positive numbers was observed in WT mice as compared to SHAM group (Figure 10b). Interestingly, TLR 7/8 KO mice showing a decreased frequency of CD3^+^ T cell as compared to WT mice (Figure 10b), reflecting an increased extravasation of T cells in the brain of these mice.

On the other hand, we also analyzed CD19^+^ as a marker of B cell. The intensity of CD19 B cell was higher in the MPTP WT mice as compared with control SHAM mice (Figure 10c). Additionally, there was a reduction in brain CD19^+^ B cell in the TLR 7/8 KO mice (Figure 10c).

### 2.10. Effect of TLR 7/8 on T Cell Production in Lymph Nodes

**Figure 11:**
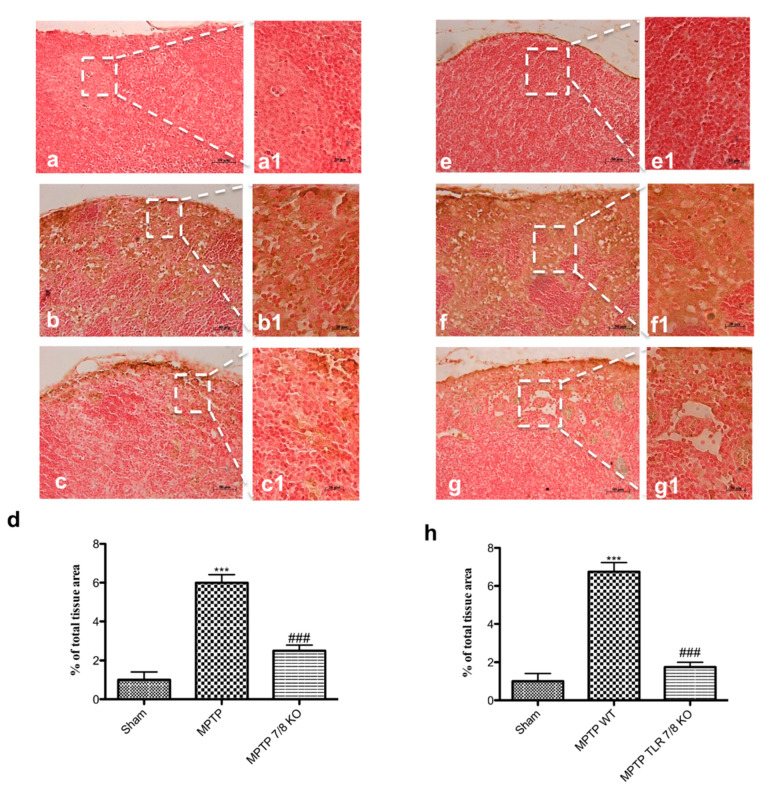
Effect of TLR7/8 on CD4^+^ and CD8^+^ expression. Immunohistochemical of CD4^+^ and CD8^+^ T cell reveal an increase for both in MPTP WT mice (**b**,**b1**,**f**,**f1**, respectively; see densitometric analysis **d**,**h**), compared to WT mice (**a**,**a1**) while the absence of TLR 7/8 markedly reduced positive staining for CD4^+^ (**c**,**c1** see see densitometric analysis **d**) and CD8^+^ T cell (**e**,**e1** see see densitometric analysis **h**) (**c**,**c1**,**g**,**g1**, respectively; see densitometric analysis **d**,**h**). Data are means ± SEM of 10 mice for each group. *** *p* < 0.001 vs. SHAM WT; ### *p* < 0.001 vs. MPTP WT.

Following a immunological stimuli, immature DCs become mature and migrate to the respective lymph node stimulating the expansion of antigen-specific T cells [2]. Thus, we evaluate the T cell production in the cervical lymph nodes by the immunohistochemistry of CD4^+^ and CD8^+^. Immunohistochemical evaluation of CD4^+^ and CD8^+^ T cell response to MPTP disclosed that both CD4^+^ and CD8^+^ positive cells were significantly increased in WT mice 7 days following MPTP injection (Figure 11(b,b1,f,f1), respectively; see densitometric analysis d,h) compared to the SHAM group (Figure 11(a,a1,e,e1), respectively; see densitometric analysis d,h). The absence of TLR 7/8 markedly reduced positive staining for both T cell antigens (Figure 11(c,c1,g,g1), respectively; see densitometric analysis d,h).

## 3. Discussion

PD is considered a multiple pathological process, including mitochondrial dysfunction, autophagy-lysosome deregulation, oxidative stress, and neuroinflammation [23,24,25]. Neuroinflammation has attracted, for a long time, the attention of scientists due to its potential in developing disease biomarkers and novel therapies [26,27]. Recently, it has been demonstrated that both innate and adaptive immunity cover an essential role in neuroinflammatory activities in PD [28,29]; in fact, previous investigations have demonstrated that adaptive immunity, particularly the T cell response, plays a central role in immune injuries in PD, additional supporting the question of whether PD is an autoimmune disorder [30,31]. The study of Koutsilieri et al. validated this thesis by studying patients with familial PD; they showed that the interaction between DCs and neuromelanin (NM), trigger the maturation of the DCs cells that subsequently migrate from the brain into the cervical lymph nodes where they present NM to B and T cells [2]. Moreover, Bandres-Ciga et al. suggested that alpha-synuclein (α-syn) is likely processed and presented by antigen-presenting cells (APCs), further activating T cells and the secretion of cytokines [32]. However, additional studies are needed to determine the molecular mechanisms underlying this autoimmune response. Innate immune cells represent a fundamental cellular mechanism mediating association among inflammatory cytokines, CNS antigens, and T-cell immunity. DCs constitute a heterogeneous subpopulation of APCs and represent the interface between foreign and tissue-specific antigens and T-cells, covering a role in all aspects of T-cell responses, from the deletion of self-reactive thymocytes to the generation of effector and memory cells and the induction of peripheral tolerance [33]. As well reported, TLRs are highly expressed on myeloid cells of the innate immune system, in particular the intracellular ones [34,35]; TLR engagement enhances antigen presentation, upregulates co-stimulatory molecule expression, and promotes the production of pro-inflammatory cytokines [36]. Various studies have suggested that direct TLR stimulation of antigen-presenting DCs is important for stimulating a strong T cell response even in the context of inflammatory cytokines from neighbouring cells [37,38]. It has been showed that TLRs may be involved in the pathogenesis of α-synucleinopathies as mediators of the non-cell-autonomous action of α-syn [39,40]. Fellner and colleagues [41] showed that the activation of glial cells by different forms of α-syn (full-length soluble, fibrillar and C-terminally truncated) was closely linked to the TLR4 and TLR2 up-regulation in mediating α-syn-induced microglial phagocytosis.

Moreover, it has been demonstrated that TLR7, TLR8, and TLR9 promote DC maturation and the production of cytokines, potentially resulting in the activation of a specific type of T-cell immunity involving the recruitment of CD8^+^ T-cells [36,42]. Thus, the first step of our work aimed to evaluate the effective role of the TLR receptors in the pathogenesis of PD following MTPT injection. The results showed a significant upregulation of TLR7 and TLR8, validating their involvement in the pathological process. It is well known that PD is characterized by a variety of behavioral deficits [43,44], resulting in a variety of symptoms concerning motor control, such as bradykinesia, rigidity, and tremor [45]. Anxiety symptoms are also very common in PD patients, and some authors state that anxiety in PD can be manifested even before the emergence of the first motor symptoms [46,47]. To validate our hypothesis of involvement of TLR7, TLR8, and TLR9, we looked at the motor and non-motor symptoms by pole test and EPM. The pole test, used to evaluate bradykinesia, showed that deletion of TLR7 and TLR8 in mice prevented the loss of locomotor agility and stability. Moreover, the absence of the genes for these two receptors also ameliorated the emotion state of mice, while knocking out the TLR9 gene did not affect any of these symptoms.

Motor and non-motor symptoms are directly linked to DA degeneration. Therefore, we evaluated the loss of TH^+^ neurons in the mesencephalon to validate the protective effect of the absence of TLR7 and TLR8 in the progression of PD. We used mice that were double-knockouts for TLR7 and TLR8 (TLR7/8 KO) to test the hypothesis that a dual antagonist of these receptors could protect from PD degeneration seen as autoimmunity disease, reducing the production of lymphocytes directed against DA neurons. We evaluated the typical PD markers, such as behavior impairment, and dopamine metabolism-related markers, including TH and DAT. Behavioral tests showed that the simultaneous absence of TLR7 and TLR8 prevented the decrease of locomotor activity following MPTP injection but did not affect the emotional behavior component. Moreover, we observed that TLR7/8 KO mice significantly preserved the levels of TH and DAT in the SNc following MPTP treatment, which is related to the ability to avoid the deterioration of DA neurons in this murine model of PD. α-syn is the main protein constituent of Lewy bodies, which are the major pathological hallmark of PD [48]. It has been noted that α-syn-derived fragments act as antigenic epitopes displayed by human leukocyte antigen (HLA) receptors. Current thinking suggests that misfolded α-syn released from neurons into the extracellular space is taken up by adjacent cells, thus leading to disease propagation [32]. Our results showed that the absence of TLR7 and TLR8 considerably reduced the α-syn aggregate formation, which can be translated into a reduction of T-cell activation. Recent research demonstrated that a T-cell response can trigger pro-inflammatory activities resulting in deregulated neuroinflammation and subsequent dopaminergic neurodegeneration. Additionally, studies have increasingly shown that therapies targeting T cells can alleviate neurodegeneration and motor behavior impairment in animal models of PD [31,49]. We looked at the expression of immune cells by flow cytometry assay and found that the frequency of CD3^+^ T cells in TLR7/8 KO mice was reduced as compared to that in WT mice, reflecting an increased extravasation of T cells in the brain of the KO mice. Moreover, T-cell accumulation is relative to DC recruitment into the sites of inflammation in the CNS during PD. The TLR7/8 KO mice clearly showed a trend of reduction of DC brain infiltration.

The auto-aggressive process of autoimmune PD may be enhanced by a triggered activation of microglia, resulting in an amplification of the adaptive immune response against NM and/or α-syn and the local reactivation of immigrating effector T cells [2]; we found that the simultaneous genetic deletion of TLR7 and TLR8 in mice significantly reduced astrogliosis and microgliosis induced in PD. Once DCs become activated (either by proinflammatory cytokines or pathogens), they migrate into the draining lymph nodes (LN) to present the antigen to naïve T and B cells [50]. The deletion of TLR7 and TLR8 significantly reduced T-cell production in the lymph nodes, suggesting a reduction of T-cell migration in the brain.

## 4. Material and Methods

### 4.1. Materials

All compounds were obtained from Sigma-Aldrich Company Ltd. (Milan, Italy). All other chemicals were of the highest commercial grade available. All stock solutions were prepared in non-pyrogenic saline (0.9% NaCl; Baxter, Rome, Italy).

### 4.2. Animals

C57BL/6 mice (male 25–30 g; 6–8 weeks old) were purchased from Charles River Italia. TLR7^-/-^, and TLR9^-/-^ single-gene-deleted mice were originally obtained from S. Akira (Osaka University, Osaka, Japan). TLR8^-/-^ knockout (KO) and TLR7/8^-/-^ double-KO mice have been described previously [51]. TLR9^-/-^ mice were a gift of Doug Golenbock (University of Massachusetts Medical School, Worcester, MA). Animals were housed in a controlled environment under conditions of controlled temperature, humidity, light (12 h light/dark cycle, lights on at 07:00 a.m.), with unrestricted access to food and water. Upon arrival, mice were left undisturbed for one week to acclimatize. This study was approved by the University of Messina Review Board for the care of animals, in compliance with Italian regulations on protection of animals (n° 537/2018-PR released on 9 July 2018 09/7/2018). Animal care was in accordance with Italian regulations on the use of animals for the experiment (D.M.116192), as well as with EEC regulations (O.J. of E.C. L 358/1 12/18/1986).

### 4.3. MPTP-Induced Parkinson’s Disease

MPTP intoxication in mice was induced by four intraperitoneal injections of MPTP (20 mg/kg; Sigma, St. Louis, MO, USA) in saline at 2 h intervals in 1 day: the total dose per mouse was 80 mg/kg. SHAM animals received vehicle only (saline). Animals were killed through isoflurane inhalation followed by decapitation 8 days after MPTP injection, and their brains were harvested, sectioned, and processed [52]. Mice were randomly allocated into the different experimental groups (*n* n = 24/each group). In a separate set of experiments with other 10 animals for each group were observed after MPTP injection in order to evaluate the behavioral testing. To minimize pain, suffering, and distress during and after the experimental procedures, all animals were observed for all the period of 7 days, as well as were administered with analgesic treatment, such as Buprenorphine treatment (0.05 mg/kg).

The dose of MPTP (80 mg/kg) used here was based on previous in vivo studies [53].

### 4.4. Experimental Groups

Animals were randomly distributed into the following 10 groups: 

Group 1: SHAM WT: vehicle solution (saline) was administered during the 1st day, like MPTP protocol, intraperitoneally.

Group 2: SHAM TLR7 KO: vehicle solution (saline) was administered during the 1st day, like MPTP protocol, intraperitoneally.

Group 3: SHAM TLR8 KO: vehicle solution (saline) was administered during the 1st day, like MPTP protocol, intraperitoneally.

Group 4: SHAM TLR9 KO: vehicle solution (saline) was administered during the 1st day, like MPTP protocol, intraperitoneally.

Group 5: SHAM TLR7/8 KO: vehicle solution (saline) was administered during the 1st day, like MPTP protocol, intraperitoneally.

Group 6: MPTP WT: MPTP solution was administered as described before.

Group 7: MPTP TLR7 KO: MPTP solution was administered as described before.

Group 8: MPTP TLR8 KO: MPTP solution was administered as described before.

Group 9: MPTP TLR9 KO: MPTP solution was administered as described before.

Group 10: MPTP TLR7/8 KO: MPTP solution was administered as described before.

In order to reach the minimum number of mice required for every technique, an ANOVA (fixed effects, omnibus, one-way) was defined “a priori” with the G-power software. This statistical test supplies a professional method to analyze the sample size required to make the experiments.

### 4.5. Behavioral Tests

Behavioral assessments on each mouse were made 1 day prior to, and 8 days after, MPTP injection. All behavioral testing was conducted during the light cycle phase and in enclosed behavior rooms (50–55 dB ambient noise) within the housing room. The mice were placed in behavior rooms for 5 min for 2 days for acclimation prior to the onset of behavioral testing. The behavioral tests were conducted by expert observers blinded to the injury status of the animals. Tests are described below. 

### 4.6. Elevated Plus Maze (EPM)

Anxiety is one of the most common neurobehavioral conditions after PD, so anxiety deficits were evaluated using Elevated Plus Maze (EPM) system at 7 days post-MPTP injection and compared with SHAM mice. We used the same methodology for EPM as the one employed in a previous study [54].

### 4.7. Pole Test

This test has been performed to detect both motor coordination and bradykinesia in PD mice [47,55]. The animals were collocated facing upwards at the top of a wooden pole (50-cm long and 1 cm in diameter) that bring into their cage. At 8 days after the final MPTP injection, the time taken for the mouse to reach the ground was recorded over 5 trials and averaged. Times were restricted to 60 s.

### 4.8. Western Blot Analysis

The brain was rapidly removed and the ventral mesencephalon was isolated by dry dissection [56]. Tissues samples from brain were processed as previously described [57].

Tissues from each mouse were suspended in extraction Buffer A (0.2 mM PMSF, 0.15 mM pepstatin A, 20 mM leupeptin, 1mM sodium orthovanadate), homogenized at the highest setting for 2 min, and centrifuged at 12,000 rpm for 4 min at 4 °C. Supernatants represented the cytosolic fraction. The pellets, containing enriched nuclei, were resuspended in Buffer B (1% Triton X-100, 150 mM NaCl, 10 mM Tris–HCl pH 7.4, 1 mM EGTA, 1mM EDTA, 0.2 mM PMSF, 20 mm leupeptin, 0.2 mM sodium orthovanadate) and centrifuged at 12,000 rpm for 10 min at 4 °C. Supernatants represented the nuclear fraction. Protein concentrations were calculated by the Bio-Rad protein assay using bovine serum albumin as standard. Briefly, samples were heated at 100 °C for 5 min, and equal amounts of protein were separated on 12% SDS-PAGE gel and transferred to nitrocellulose membrane. Specific primary antibody, TLR7 (1:500 Santa Cruz Biotechnology, sc 57463, RRID:AB_793196, California CA, USA), anti-TLR8 (1:500 Santa Cruz Biotechnology, sc 25467, RRID:AB_2203501, CA, USA), anti-TLR9 (1:500 Santa Cruz Biotechnology, sc 52966, RRID:AB_793207, CA, USA), anti- Glial fibrillary acidic protein (GFAP) (1:500; Santa Cruz Biotechnology, 9065, RRID:AB_641022, CA, USA), anti- Ionized calcium binding adaptor molecule 1 (IBA1) (1:500; Santa Cruz Biotechnology, sc- 32725, RRID:AB_667733, CA, USA), anti-αSyn (1:500; Santa Cruz Biotechnology, sc 69977, RRID:AB_1118910, CA, USA), anti-Tumor Necrosis Factor (TNF) α (1:500; Santa Cruz Biotechnology, sc 1350, RRID:AB_2204365, CA, USA) were mixed in 1× PBS, 5% w/v nonfat dried milk, 0.1% Tween-20 (PMT) and incubated at 4 °C, overnight. After, membranes were incubated with peroxidase-conjugated bovine anti mouse IgG secondary antibody or peroxidase-conjugated goat anti rabbit IgG (1:2000, Jackson ImmunoResearch) for 1 h at room temperature. To ascertain that blots were loaded with equal amounts of proteinic lysates, they were also incubated in the presence of the antibody against β-actin (1:5000; Santa Cruz Biotechnology) for cytosolic proteins.

Signals were detected by enhanced chemiluminescence (ECL) detection system reagent according to the manufacturer’s instructions (SuperSignal West Pico Chemiluminescent Substrate, Thermo Fisher Scientific, Waltham, MA, USA). The relative expression of the protein bands was quantified by densitometry with BIORAD ChemiDoc^TM^XRS+software and standardized to β-actin levels. Images of blot signals (8 bit/600 dpi resolution) were imported to analysis software (Image Quant TL, v2003).

### 4.9. Immunohistochemical Localization of TH, DAT, CD4^+^, and CD8^+^

We used the same methodology for immunohistochemical localization as the one employed in a previous study [58]. Briefly, sections (7 μm) were prepared from paraffin embedded tissues. After deparaffinization, endogenous peroxidase was quenched with 0.3% hydrogen peroxide in 60% methanol for 30 min. The sections were permeabilized with 0.1% Triton X-100 in PBS for 20 min. Sections were incubated in a humidified chamber overnight at 37 °C with anti-Tyrosine hydroxylase (TH) polyclonal antibody (1:250, Millipore), anti-dopamine transporter (DAT) antibody (1:100, Santa Cruz Biotechnology, sc 14002, CA, USA), anti-CD4^+^ (1:500, Santa Cruz Biotechnology, sc 52480, CA, USA), and anti-CD8^+^ (1:500, Santa Cruz Biotechnology, sc 7970, CA, USA). Sections were washed with PBS and incubated with secondary antibody. Specific labeling was detected with a biotin-conjugated goat anti-rabbit IgG and avidin-biotin peroxidase complex (Vector Lab, Inc., Burlingame, CA, USA). To verify antibody-binding specificity, control slices were incubated with only primary antibody or secondary antibody, neither of which gave positive staining.

Images were collected using a Zeiss microscope and Axio Vision software. For graphic display of densitometric analyses, the % of positive staining (brown staining) was measured by computer-assisted color image analysis (Leica QWin V3, UK). The percentage area of immunoreactivity (determined by the number of positive pixels) was expressed as % of total tissue area (red staining) within five random fields at ×40 magnification. In particular, firstly, the colors of the images that have been stained to the molecule of interest were defined. Once these colors were defined, they were automatically detected in all samples. This is a semi-quantitative analysis that measures areas and not intensities [59,60].

### 4.10. Immunofluorescence Staining

Sections were processed for immunofluorescence staining as previously described [55]. Sections were incubated with mouse monoclonal anti-TH (1:100, *v/v* Millipore), or with polyclonal rabbit anti- αSyn (1:100, *v/v* Santa Cruz, Biotechnology, CA, USA) antibody in a humidified chamber for O/N at 37 °C. Sections were observed and photographed at ×100 magnification using a Leica DM2000 microscope (Leica, UK, EU). All images were digitalized at a resolution of 8 bits into an array of 2560 × 1920 pixels. Optical sections of fluorescence specimens were obtained using a HeNe laser (543 nm), a laser UV (361–365 nm) and an argon laser (458 nm) at a 1 min, 2 s scanning speed with up to 8 averages; 1.5 μm sections were obtained using a pinhole of 250. Contrast and brightness were established by examining the most brightly labeled pixels and applying settings that allowed clear visualization of structural details while keeping the highest pixel intensities close to 200. The same settings were used for all images obtained from the other samples that had been processed in parallel. Digital images were cropped and figure montages prepared using Adobe Photoshop CS5 (Adobe Systems; Palo Alto, CA, USA). Cell counting analysis was made on rostro-caudal brain slices for a total of three slices per animal (n = 10 for each group).

### 4.11. Flow Cytometry

Flow cytometry was performed as previously described [61]. The brain tissues were diced in plastic weigh boats with a razor blade and mechanically homogenized. Subsequently, tissues were torn apart in sterile PBS by mechanical pressure through a 70 μm mesh cell strainer (Falcon). Leukocytes were isolated by density gradient centrifugation in Percoll (GE Healthcare, Rome Italy). In detail, cells were pelleted at 400× *g* for 5 min and suspended in a 30% Percoll solution. The 30% Percoll solution was carefully layered onto a 70% Percoll solution in a ratio 1:2 and centrifuged at 500× *g* for 30 min. In this density, gradient mononuclear cells sediment at the interface between 30% and 70% Percoll layers. About 2–3 mL of interface solution was collected only after the fatty layer at the top of the centrifuge tube was carefully removed. Then, the purified mononuclear cells were washed twice in RPMI supplemented with 100 U/mL of penicillin and streptomycin and then suspended in FACS buffer (phosphate-buffered saline containing 5% fetal calf serum and 0.02% of NaN2) for further analysis. Aliquots of cell suspensions were used to evaluate the total number of cells using a standard hematocytometer. Cells were stained in 100 μL of FACS buffer containing the following fluorochrome-conjugated antibodies: CD3 (1:100 FITC Rat anti-mouse clone 17A2 # 100203 Biolegend), CD19 (1:100 PE Rat anti-mouse clone 1D3 # 553786 BD Pharmigen), and CD11c (1:100 PerCP anti-mouse clone 17A2 #117325 Biolegend) and the respective isotype antibodies as controls. Cells were labeled with the appropriate concentration of conjugated antibodies for 1 h at 4 °C. Samples were analyzed on a FACSCanto II flow cytometer with the FlowJo software (both from BD Bio-sciences).

### 4.12. Statistical Evaluation

All values in the figures and text are expressed as mean ± standard error of the mean (SEM) of N observations. For the in vivo studies, N represents the number of animals studied. In the experiments involving histology, the figures shown are representative of at least three experiments (histological coloration) performed on different experimental days on the tissue sections collected from all the animals in each group. The results were analyzed by one-way ANOVA followed by a Bonferroni post-hoc test for multiple comparisons. A *p*-value of less than 0.05 was considered significant.

## 5. Conclusions

In conclusion the object of the present study was to identify new therapeutic target for the modulation of PD pathogenesis. Specifically, we targeted activation and/or modification of the neuroinflammatory process and immune response related to PD, since these are principal events in neurodegenerative pathogenesis. The gap between the neuroinflammation and immune response could be TLRs 7 and 8 that represents an important promoter of a T cells response, even in the context of inflammatory cytokines. In particular, we speculated that the autoimmune process in PD is unleashed by α-syn fragments binding to TLR 7 and 8, considered as antigenic epitopes, on DC. This process lead to the amplification of adaptive immune response against α-syn.

Our findings raise the possibility that an immunotherapy approach, by using a dual antagonist of TLR 7 and 8, could be used to increase the immune system’s tolerance for α-syn or other miss-folded proteins, which could help to ameliorate or prevent worsening symptoms in PD patients.

## Data Availability

The datasets used and/or analyzed during the current study are available from the corresponding author on reasonable request.

## Abbreviations

APC	antigen-presenting cells
DAT	dopamine transporter
EPM	Elevated plus maze
GFAP	Glial fibrillary acidic protein
IBA1	Ionized calcium binding adaptor molecule 1
LN	lymph nodes
LPS	lipopolysaccharides
mDCs	monocyte-derived dendritic cells
MPTP	1-metil 4-fenil 1,2,3,6-tetraidro-piridina
NM	neuromelanin
PAMPs	Pathogen Associated Molecular Patterns
PD	Parkinson Disease
pDC	plasmocytic dendritic cells
RRIDs	Research Resource Identifiers
SLE	systemic lupus erythematosus
TH	Tyrosine hydroxylase
TLR-7 KO	Toll-like receptor 7 knock out
TLR-7/8 KO	Toll-like receptor 7/8 knock out
TLR-7/9 KO	Toll-like receptor 7/9 knock out
TLR-8 KO	Toll-like receptor 8 knock out
TLR-9 KO	Toll-like receptor 9 knock out
TLRs	Toll-like receptors
TNFα	Tumor Necrosis Factor α
α-syn	α-Synuclein
